# Report on Witteveen-Kolk syndrome caused by large fragment deletion in the 15q24.1 - q24.2 region in infants with early onset and literature review

**DOI:** 10.1186/s13052-025-01971-3

**Published:** 2025-05-07

**Authors:** Hanlin Tang, Jiaxuan Xu, Ling Ge, Qunting Gao, Xinlu Tan, Qicheng Qiao, Ruihan Liu, Qingxia Kong, Qiubo Li, Xiufang Jiang

**Affiliations:** 1 Jining Medical University, Jining, Shandong 272029 China; 2 Shandong Provincial Key Medical and Health Discipline of Pediatric Internal Medicine (Affiliated Hospital of Jining Medical University), Jining, Shandong 272029 China; 3 Jining Key Laboratory for Prevention and Treatment of Severe Infection in Children, Jining, Shandong 272029 China; 4 Affiliated Hospital of Jining Medical University, Jining, Shandong 272029 China

## Abstract

**Background:**

WITKOS is a rare neurodevelopmental disorder caused by heterozygous loss-of-function variants in the 15q24.1 - q24.2 region, includeing switch-insensitive 3 transcription regulator family member A (SIN3 A). Its incidence rate is extremely low. According to the current limited global data statistics, only a few cases occur per million population. Most cases concentrate in childhood and adolescence. During this stage, the rapid physiological changes of the body seem to be closely related to the triggering of the disease. Its characteristics include unique facial features, intellectual and motor developmental delay, and short stature. This paper reports a case of WITKOS in a 4-month-old infant caused by large-fragment copy number variation in the 15q24.1 - q24.2 region of the chromosome that encompasses the SIN3 A gene. Gene mutations lead to abnormal functions of key proteins, which subsequently disrupt the normal development and operation of multiple body systems., By summarizing its gene phenotype characteristics, it provides diagnostic and treatment ideas for clinicians to screen for developmentally deviated young children. It is expected to provide more effective treatment options for WITKOS to improve the prognosis of patients.

**Case presentation:**

This infant was diagnosed with WITKOS at 4 months of age. Subsequently, the manifestations included a wide forehead, a low nasal bridge, low-set ears, growth and intellectual developmental delay, low muscle tone in the limbs, and feeding difficulties. After early rehabilitation training, the language and motor abilities of this infant have been effectively improved, and currently, the infant can walk and run independently and can say short sentences independently.

**Conclusions:**

For children with WITKOS, early diagnosis of the clinical symptoms they exhibit and rehabilitation intervention should be carried out, which can effectively improve the quality of life of the children. This report is the first case of WITKOS caused by a large - fragment deletion, further enriching the case data of WITKOS and highlighting the necessity of strengthening clinical management and monitoring.

## Background

WITKOS is a rare neurodevelopmental disorder that was first described in 2016, with distinctive facial features (such as a broad and high forehead, small mouth, thin upper lip, pointed chin, and downward-slanting palpebral fissures), microcephaly, short stature, mild intellectual disability (ID), cognitive and motor developmental delays, and corpus callosum dysplasia and ventricular enlargement on brain Magnetic Resonance Imaging (MRI), etc. [1].The disease is caused by SIN3 A heterozygous loss-of-function variations and is also characterized by various intellectual disabilities and developmental delays. WITKOS is inherited in an autosomal dominant manner, and most patients are sporadic. In terms of the existing literature, up to now, the reported research has mainly focused on the patient groups of older children, adolescents, and adults. There has been no relevant report on the case of WITKOS caused by large - fragment deletion in infancy. This report aims to fill this gap and expand the case data of WITKOS. This study reports a case of WITKOS caused by copy number variation of SIN3 A in a 4-month-old infant, summarizes the characteristics of its gene phenotype, and provides some diagnostic and therapeutic ideas for clinicians in screening for developmentally deviated young children. It is hoped that through this study, more effective treatment options for WITKOS can be provided to improve the prognosis of patients.

## Case presentation

**Fig. 1:**
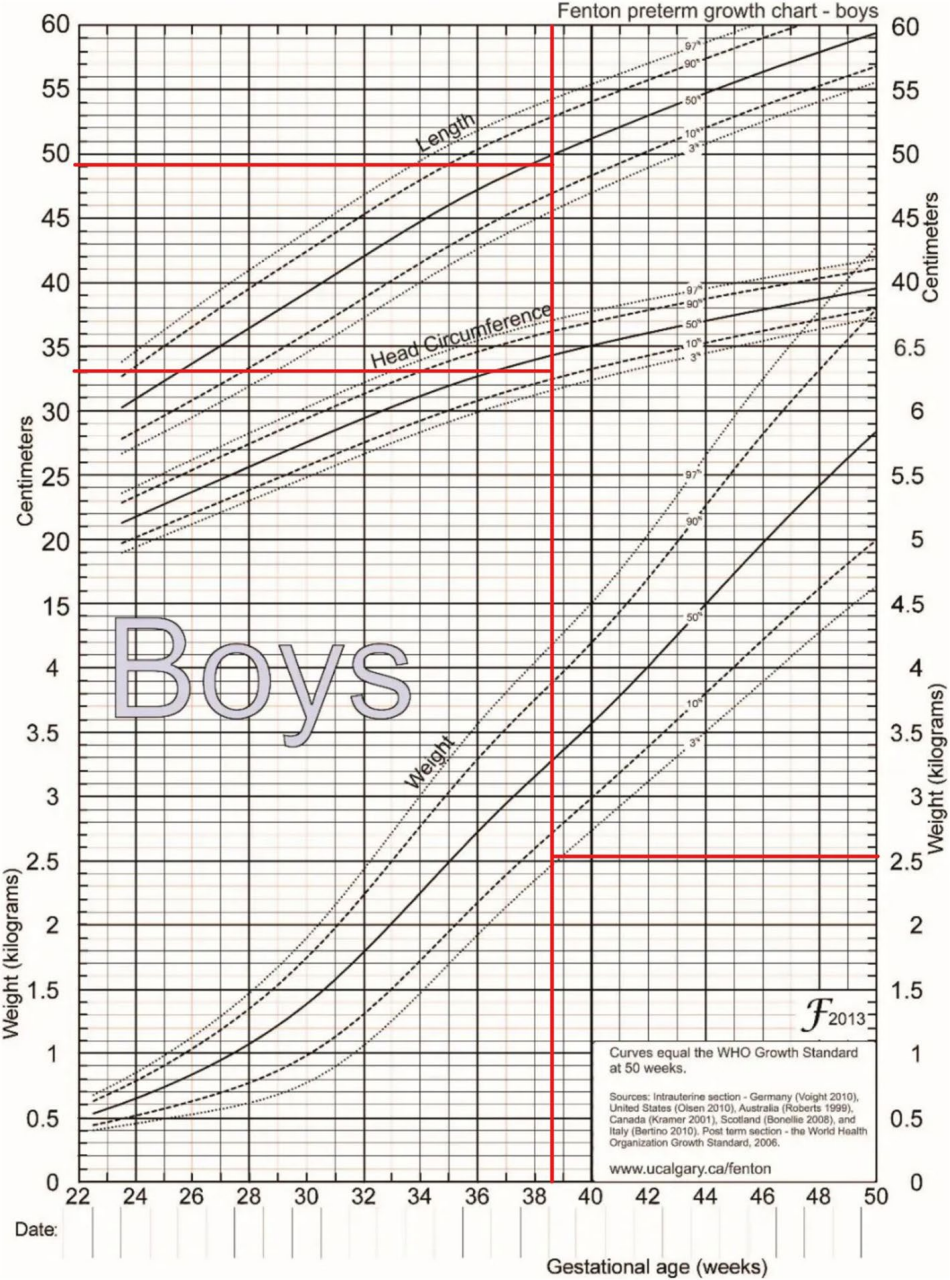
Schematic diagram of the FENTON curve chart of the patient, and the intersection point represents the patient's situation

**Fig. 2:**
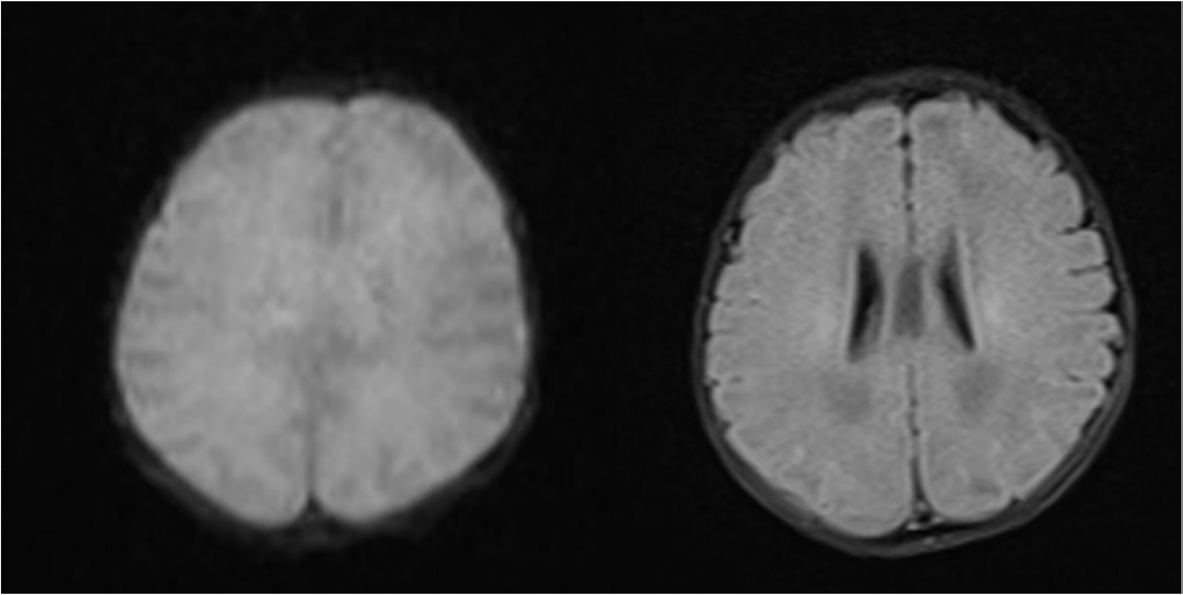
Brain MRI plain scan and diffusion-weighted imaging of the patient

**Fig. 3:**
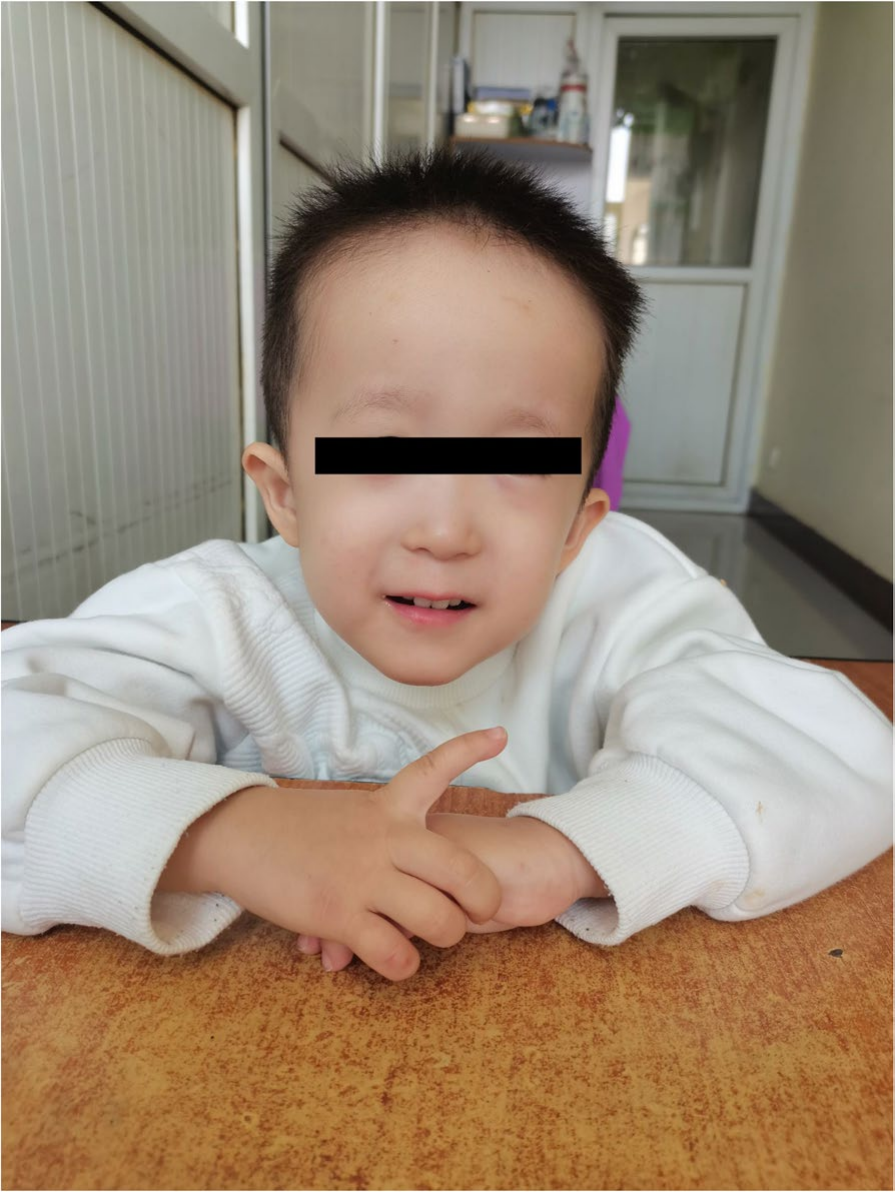
The facial features of this child: a wide and high forehead, a low nose bridge, full and drooping eyelids, small lips, a small mandible, and a low ear position

**Fig. 4:**
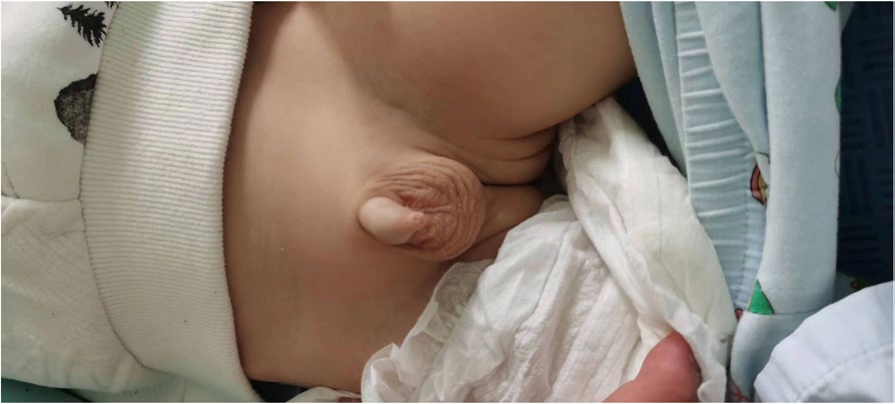
It can be seen that the cryptorchidism of the child has been effectively improved

The patient was a 4-month and 2-day-old male infant who presented with"unstable neck holding"on November 12, 2021. Physical examination showed a weight of 6.0 kg (−1 to −2SD), a height of 60.1 cm (−2SD), and a head circumference of 41.7 cm (0SD). The special facial features included a wide and high forehead, a low nasal bridge, full and downward-slanting eyelids, a small mouth, a small mandible, and low-set ears. There were no abnormalities in the heart and lungs. Genital examination revealed a short penis and left cryptorchidism. Motor examination showed that the muscle tone of the limbs was slightly low during the first physical examination, but on December 17, 2021, there was a decrease in muscle tone, a positive scarf sign, poor head control, head retroflexion when pulled up from the supine position, and unstable neck holding, but the ability to lift the head was present. Both hands could be centered and could suck the hands. Cognitive and reactive language examination showed few facial expressions, little interaction, poor repetitive visual tracking, rarely crying, few babbling sounds, inability to be amused to laugh, and no awareness of grasping objects. The child was the second pregnancy and second birth (G2P2). The mother underwent cesarean section at 38 + 5 weeks of gestation due to"polyhydramnios, pregnancy with uterine scar, vaginitis, and mild anemia"during pregnancy. At birth, the weight was 2.53 kg (3.8%), the height was 49.0 cm (33.7%), the head circumference was 33.0 cm (17.5%), and the Apgar score was 10 at 1, 5, and 10 min, respectively, indicating a small-for-gestational-age infant (see the FENTON curve chart, Fig. 1). After birth, the child was fed with a mixed diet and had difficulty in feeding, with each breastfeeding session lasting more than half an hour, and the growth rate was slow. On the first day after birth, the child was hospitalized in the NICU due to"neonatal amniotic fluid swallowing syndrome and neonatal ABO blood group incompatibility hemolytic disease", and the jaundice reached a maximum of 17.0 + mg/dl. On the third day of hospitalization, brain MRI plain scan and diffusion-weighted imaging showed no obvious abnormalities (Fig. 2), and the child was discharged after improvement three days later. On July 27, 2021, the thyroid function test was normal. On November 11, 2021, during the 4-month physical examination, the developmental test score was 62, the bone density was − 0.8, and the neuropsychological developmental test for children aged 0–6 years showed a developmental quotient of 62 (the normal value is between 85 and 115), with a gross motor score of 37, fine motor score of 74, adaptive ability score of 50, language score of 87, and social behavior score of 62. After diagnosis, the child received early rehabilitation intervention locally. On October 6, 2022, during a telephone follow-up at 15 months of age, the child's height was about 80 cm and the weight was about 10 kg. The progress of gross motor skills was faster than that in other functional areas. The child could not walk independently but could stand with support for a short time, could walk and bend over to pick up objects while supported, could get on and off the bed independently, and could play with toys independently. The muscle tone was low, the child could hold the bottle to drink milk, and the femoral angle was 180°. The progress of fine motor skills, cognition, and language was slower, there were unconscious slapping movements, the child could not understand one-step instructions, but could call mom and dad. The father's blood type was A, Rh unknown, and he was healthy. The mother's blood type was O, Rh positive, and both parents had medium stature. They were not closely related, and had an older sister who was 3 years and 8 months old. The sister was hospitalized in the neonatal intensive care unit of the local hospital for 10 days after birth and is currently healthy and developing normally, with no similar family history.In the follow-up on October 12, 2024, the child can currently walk and run independently and can say short sentences but not long ones, After rehabilitation intervention, the overall development score of the child has increased from the initial 217 points to 278 points (sensory perception 15 - 22, gross motor skills 79 - 91, fine motor skills 22 - 28, self - care ability in daily life 42 - 50, language communication 36 - 45, cognition 11 - 19, sociality 12 - 23), and the overall improvement effect is good. Figure 3 shows the photo of the child during the follow-up. It can be seen that the child still has a wide and high forehead, a low nose bridge, full and drooping eyelids, small lips, a small mandible, and a low ear position. The cryptorchidism of the child has been effectively improved. At present, no obvious abnormal manifestations have been observed. See Fig. 4.

### Chromosome examination

**Fig. 5:**
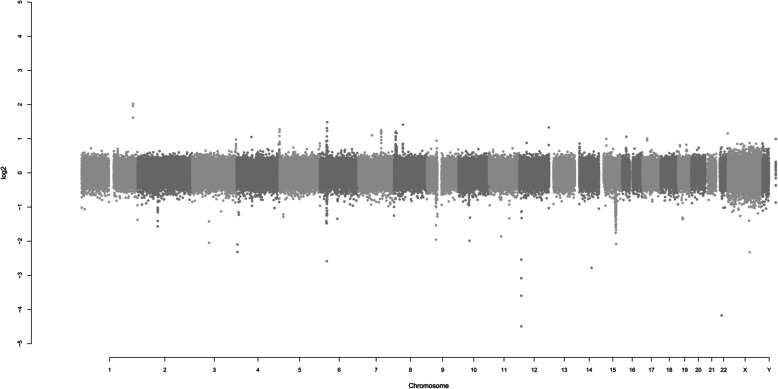
Genome-wide detection result diagram of the examined patient: The composition of the examined chromosomes is 46, XY, and the number of chromosomes is normal, This information provides a basis for subsequent analysis and rules out the possibility that the disease is caused by abnormal chromosome numbers

After obtaining informed consent from the parents of the patient, blood samples were collected for chromosome aneuploidy and microdeletion or microduplication examination. The report date was December 09, 2021, and the examination results were as follows:

Chromosome aneuploidy: The composition of the examined chromosomes was 46, XY, and the number of chromosomes was normal. See Fig. 5. Chromosome microdeletion or microduplication: CNV detection revealed pathogenic CNV variations. Through copy number variation verification (qPCR - SYBR Green I dye method), a deletion variation was found in one region. Interpretation of the abnormal result: Abnormal type: deletion (loss).

**Fig. 6:**
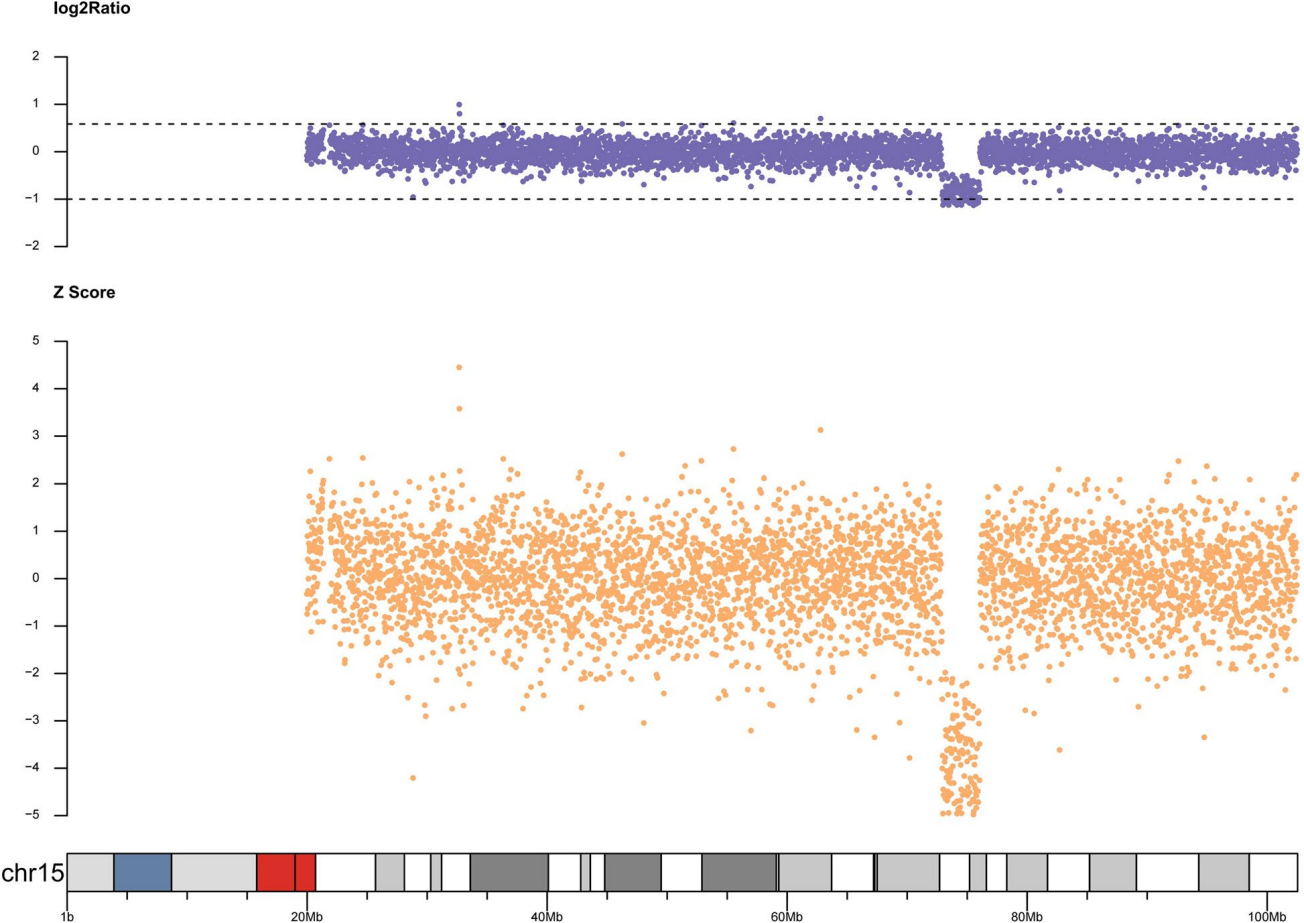
Copy number distribution map of the examined patient on chr15 chromosome. Chromosome Location: 15q24.1-q24.2 Start-End Position of the Abnormal Fragment (UCSC hg19): 72,929,607 - 76,069,619 Size of the Abnormal Fragment (Mb): 3.140, This change in copy number is closely related to the patient's disease state and may be an important genetic factor leading to the symptoms of WITKOS

Pathogenicity: Pathogenic. Chromosome location: 15q24.1 - q24.2. Start - end position of the abnormal fragment (UCSC hg19): 72,929,607 - 76,069,619. Size of the abnormal fragment (Mb): 3.140. The copy number distribution of the examined person on chr15 is shown in Fig. 6.

**Fig. 7:**
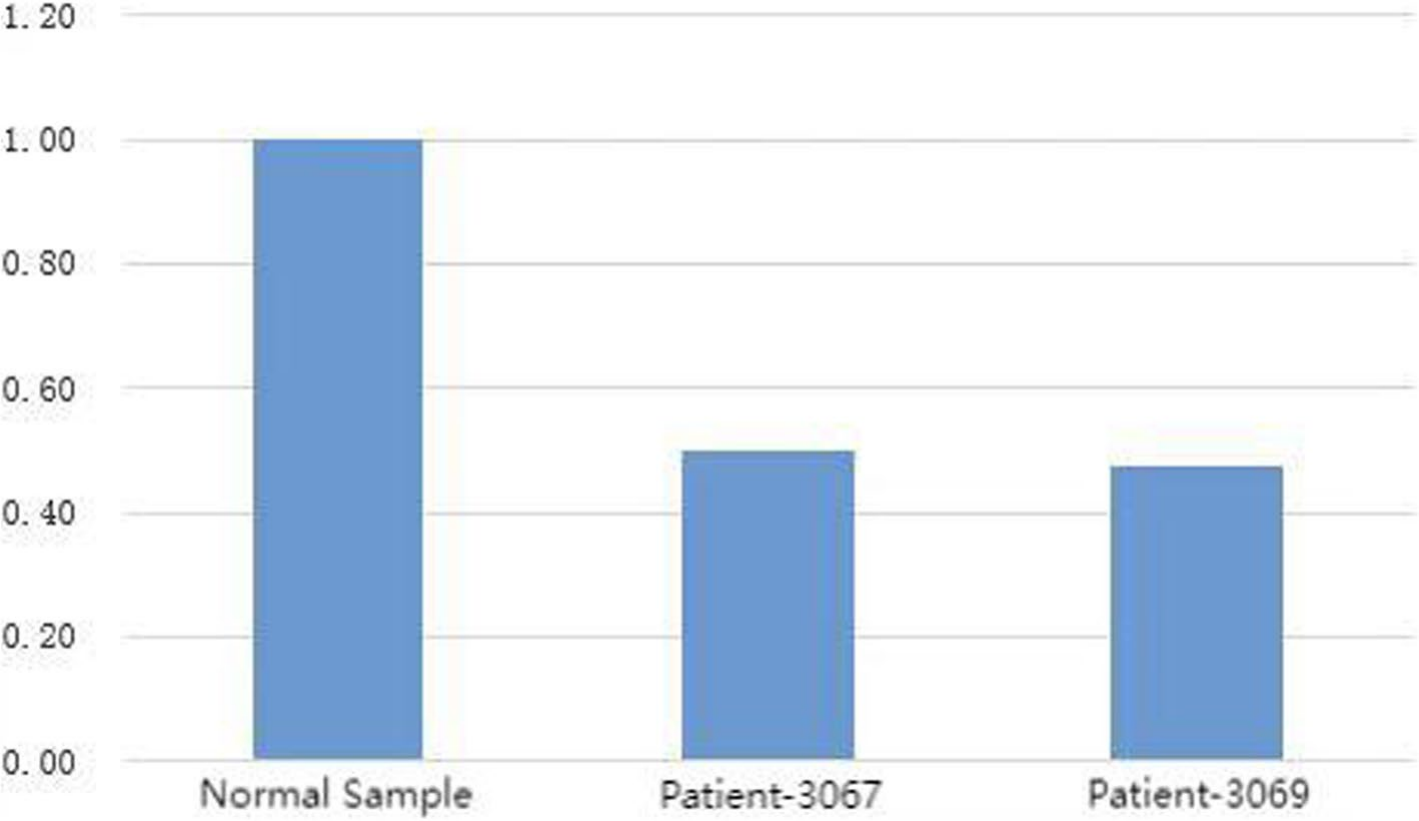
QPCR verification result. Note: The chromosome ranges verified by primers 3067 and 3069 are both partial segments of the detected result chr15: 72,929,607 - 76,069,619. Primer 3067 sequence: Forward: TGTTAGGGGCTTCTCGGGAT; Reverse: TCAGGGGGAGGATTAGGGTC. Primer 3069 sequence: Forward: GCAGTCCCCCTAAGTCCAAG; Reverse: AGAGAATCTGGTGCAGTCGC

Using fluorescence quantitative PCR to analyze the data further, we selected suspicious fragments similar to the proband's phenotype and then did Q - PCR experiments to verify the region, excluding false positives of second-generation sequencing and ensuring result accuracy. The specific experimental process is as follows: The experimental instrument used was the 7300Plus Real - Time PCR System, and the reagent used was UltraSYBR Mixture (High ROX). For the PCR system, the reaction mixture included 12.5 μl of enzyme, 10.5 μl of water, 1 μl of genome (with a concentration of 50 ng/μl), and 1 μl of primer (with a concentration of 5 μmol/L). The temperature settings were as follows: an initial step at 95 °C for 10 min, then 38 cycles with each cycle including steps at 95 °C for 15 s, 63 °C for 45 s (with fluorescence detection), then again at 95 °C for 15 s, and 63 °C for 1 min (with fluorescence detection during heating), and after the cycles, additional steps at 95 °C for 15 s and 63 °C for 15 s. The analysis software used was Real - Time PCR Software V2.4, and the final result was obtained. The QPCR verification result is shown in Fig. 7. The main purpose of primer design is to specifically recognize the target DNA sequence and guide DNA polymerase to carry out amplification reactions under appropriate conditions. Primers are short single-stranded DNA sequences that can pair complementarily with specific regions on the template DNA, thereby determining the starting position of amplification.

The whole exome sequencing analysis of the child showed that no pathogenic or suspected pathogenic variants that could explain the phenotype of this child were found.

The result of the Q - PCR experiment on the detection result was that the copy number was approximately 0.5, and the copy number of a normal sample (diploid) was 1. The experimental result indicated that this patient indeed had a copy number abnormality at 15q24.1 - q24.2. Based on the clinical characteristics of this patient and the examination results, WITKOS was finally diagnosed. Moreover, there is a likelihood that this case may have another coexisting genetic disease, which might also be one of the reasons for the phenotype of the child reported in this paper.

## Discussion and conclusions

The genetic analysis of the infant in this study revealed that within the 15q24.1 - q24.2 chromosomal region, several genes were identified, with SIN3 A being the key pathogenic gene. Other genes such as CYP11 A1, STRA6, PML, HCN4, LOXL1, and MPI were also present, although their direct relevance remains to be further explored. The related pathogenic gene with the same dominant inheritance pattern as SIN3 A is HCN4. Mutations in the HCN4 gene can manifest as cardiovascular symptoms and language developmental delay. The child has not been detected with reduced cardiovascular system function but shows language developmental delay, which may contribute to the phenotype of this child. According to the Clingen report, SIN3 A's haploid dose score is 3. Enough evidence shows dose sensitivity is related to the phenotype. So, the deletion in the 15q24.1 - q24.2 region including SIN3 A may have caused the child's phenotype.

We searched but didn't find prior reports of WITKOS due to large - fragment deletions like ours. This offers a new perspective on how such gene changes impact WITKOS symptoms. This child in our report did not show seizures, abnormal vision and hearing, abnormal brain MRI, or behavioral problems. (Table 1) However, most children with WITKOS caused by other single-gene abnormalities have these abnormalities. We wonder if the loss of large gene fragments has led to the non-appearance of these abnormalities. In the follow-up, we need to pay close attention. At the same time, more cases of large gene fragment deletions are needed to prove whether our conjecture is correct.

**Table 1:** Comparison of the characteristics of this patient with reported patient

	Demographic Data			Genetic Data		Medical Characteristics				
	Male	Female	Age	Chromosome	Mutation	Specific Facial Features(broad forehead, small mouth, etc.)	Small—for—Gestational—Age Infant	Intellectual Disability or Developmental Delay	Feeding Difficulties	Decreased Muscle Tone
This study (*n* = 1)*	1	-	4 months and 2 days	Chr15: 72,929,607–76069619	NR	1	1	1	1	1
Narumi-Kishimoto Y (*n* = 1) [2]	-	1	7 years	NR	c.848dup	1	1	1	NR	-
Balasubramanian M et al. (*n* = 28) [3]	17	11	Average 8.2 years	NR	1.c.1462del,p.(Val488Leufs*7) 2.c.2764C > T,p.(Arg922*) 3.c.588del,p.(Asn197Metfs*4) 4.c.377C > T,p.(Ala126Val) 5.c.1245_1246del,p.(Asn415Lysfs*24) 6.c.775dup,p.(His259Profs*47) 7.c.824del,p.(Pro275Hisfs*12) 8.c.2248_2251del,p.(Leu750Metfs*43) 9.c.2339del,p.(Ala780Glyfs*14) 10.c.889C > T,p.(Gln297*) 11.c.1715_1722delinsCCCAAGTGTA, p.(Gly572Alafs*11) 12.c.3323C > G,p.(Ser1108*) 13.c.3490A > T,p.(Lys1164*) 14.c.46C > T,p.(Gln16*) 15.c.3310C > T,p.(Arg1104*) 16.c.2809_2810del,p.(Lys937Glufs*2) 17.c.1489_1499del,p.(Arg497Cysfs*13) 18.c.3317 T > C,p.(Met1106Thr) 19.c.1675C > T,p.(Arg559*) 20.c.3303C > G,p.(Tyr1101*) 21.c.1570_1577del,p.(Tyr524Valfs*26) 22.c.2185_2186del,p.(Leu729Glyfs*8) 23.c.3441_3445del,p.(Lys1148Argfs*12) 24.c.1318_1737dup;p.(Val580Lysfs*35) 25.c.1773G > A,p.(Trp591*) 26.c.463A > G,p.(Lys155Glu) 27.c.2803C > T,p.(Arg935*) 28.c.1888dup,p.(Ile630Asnfs*17)	28	NR	13	15	12
WitteveenJS et al. (*n* = 15) [1]	7	7 (1 person unknown)	4–45 years	Chr15	1. Del 75.60–76.10 2. Del 75.60–76.10 3. Del 75.60–76.02 4. Del 75.60–75.95 5. c.803dup; p.Leu269fs 6. c.1010_1013del; p.Lys337Serfs 7–9. c.1759_1759delT; p.Ser587fs 10. c.2955_2956del; p.Glu985fs 11–13. c.3310C > T; p.Arg1104 14. Del 75.53–75.80 15. Del 75.59–76.09	15	NR	12	NR	2
Adam Jacobson, MD et al. (n = 2) [4]	-	2	7 years 2 years	NR	c.1657C > T, p.R553	2	NR	2	NR	NR
Linde C. M. van Dongen et al. (*n* = 5) [5]	3	2	14 years 10 years 12 years 23 years 10 years	Chr15(GRCh37)	1.c.1010_1013del, 2.c.2955_2956del, 3.c.803dup, 4.c.1675C > T, 5.c.1488_1498del	NR	NR	5	NR	NR
Total (*n* = 52)	28	23				47	2	34	16	15

	Medical characteristics						
	Seizures	Behavioral Problems	Short Stature	Microcephaly	Vision Abnormalities	Hearing Abnormalities	Brain MRI Abnormalities
This study (*n* = 1)*	-	-	1	-	-	-	-
Narumi-Kishimoto Y (*n* = 1) [2]	-	1	-	-	NR	-	NR
Balasubramanian M et al. (n = 28) [3]	4	12	6	4	4	6	4
WitteveenJS et al. (* n* = 15) [1]	3	6	7	6	NR	4	7
Adam Jacobson, MD et al. (*n* = 2) [4]	NR	NR	NR	NR	2	NR	NR
Linde C. M. van Dongen et al. (*n *= 5) [5]	3	5	NR	NR	2	4	3
Total (*n* = 52)	10	24	14	10	8	14	14

*NR* Not Reported

^*^In this case, the infant did not present with seizures, abnormal vision and hearing, abnormal brain MRI findings, or behavioral problems

In mammals, the eukaryotic chromatin structure is made up of histone octamers of the core histones H2 A, H2B, H3, and H4, which form nucleosomes to ensure that the DNA is compressed. Post-translational modification of the N-terminus of histones, especially acetylation, plays a decisive role in the dynamic regulation of gene transcription. histone deacetylation is performed by histone deacetylase (HDAC) HDAC1 and HDAC2 and is dependent on complexes containing Sin3, MecP2, NuRD, and CoREST. Recruitment of the Sin3 corepressor module leads to deacetylation of histones H3 and H4. SIN3 A encodes a transcriptional regulatory protein associated with scaffold formation in core histone deacetylase complexes [6]. The formed SIN3-HDAC complex serves as a cofactor scaffold and promotes the histone H3 and H4 deacetylation process by recruiting Sin3 corepressor modules [6–8]. liu and pile et al. [9] believe that SIN3 is an important epigenetic regulator and is directly linked to methionine metabolism and histone modification. SIN3 A plays an integral role in the development of the nervous system. SIN3 A is required for cortical progenitor cell differentiation, and decreased SIN3 A expression will lead to altered cortical neuron identity. In addition, SIN3 A expression also affects cortical differentiation and axon elongation. Downregulation of SIN3 A expression increases the length and number of axons that cross the midline along the corpus callosum pathway, while decreased expression results in axon elongation deviating from the original route. Functional knockout of SIN3 A in vivo resulted in reduced neurogenesis, altered neuronal identity, and abnormal cortical projection in developing mice. In mammals, there are two genes responsible for encoding the main factors of the Sin3 complex, namely SIN3 A and SIN3B [10]. If SIN3 A is absent or has other abnormalities, WITKOS with special facial features and clinical symptoms such as intellectual impairment may occur.

The SIN3 A gene is in the 15q24 segment and is a key part of the transcriptional corepressor complex. It was once seen as important for the 15q24 microdeletion syndrome [11]. The Sin3 A transcription protein, with a molecular scaffold function, offers a platform for functional transcription factors. It has six domains, including a histone interaction domain (HID) that can recruit core complexes like HDAC, histone-binding protein RBAP46/48, stabilizer SAP18/20, and SDS3 [12]. Sin3 A is crucial for transcription regulatory factors like HDAC [1]. It's one of the main genes encoding the Sin3 complex, and its encoded protein is related to various physiological processes. In nervous system development, SIN3 A is essential. WITK0S results from SIN3 A function loss, and its haploinsufficiency causes WITKOS [1, 3]. Regarding SIN3 A's effect on patient methylation status, studies have different conclusions. Suzuki et al. [13] and Liu & Pile [9] think its haploinsufficiency may affect WITKOS patients'methylation. Kawai [14] found no methylation difference. Narumi-Kishimoto Y [2] detected no abnormal DNA methylation, believing SIN3 A frameshift mutations don't impact it. Jet Coenen - van der Spek [15] established a WITKOS DNA methylation epigenetic probe, diagnosed an undiagnosed case, and suggested SIN3 A haploinsufficiency may not be the only factor.

Witteveen et al. [1] reported that some WITKOS patients had motor disorders (gross and fine motor skills), which is a developmental coordination disorder (DCD) diagnosed by motor coordination difficulties. DCD also exists in other neurodevelopmental disorder children, so it's often not recognized and diagnosed by medical professionals [16]. The patient in this report, like Narumi-Kishimoto Y [2]'s case, was a small-for-gestational-age infant without microcephaly, vision/hearing abnormalities, brain MRI abnormalities, or seizures. Table 1 [1–5] summarized reported WITKOS patients'characteristics. This child was diagnosed after 4 months with intellectual developmental delay (poor cognitive understanding, no active language), motor developmental delay (decreased muscle tone, delays in gross and fine motor skills), short stature, and special facial features. After early rehabilitation training, the child's motor ability improved. Clinically, similar patients need early assessment and rehabilitation. For those with early feeding difficulties, provide feeding guidance and observe growth. Identify and manage behavioral problems early and follow up for ASD. The disease has a poor prognosis and requires long-term intervention. Despite its low incidence and sporadic nature, due to its autosomal dominant inheritance, parents of an affected child should be examined. If a parent has a 15q24 microdeletion or SIN3 A gene abnormality, the offspring has a 50% inheritance chance. Third-generation in vitro fertilization technology (PGD) is recommended for intervention. Future research should focus on SIN3 A function, treatment methods, and improving patient management and awareness. Comparing with 15q24 microdeletion syndrome children, this WITKOS child has similar growth, intellectual, facial, and muscle tone characteristics, making clinical distinction difficult and highlighting the need for gene testing.

Our WITKOS patient and children with 15q24 microdeletion syndrome have some similar characteristics. Magoulas PL [17] found that SIN3 A gene problems can cause special symptoms, which means SIN3 A is important. Both diseases have growth retardation, abnormal intellectual development, special facial features, and abnormal muscle tone. These similarities make it hard to tell them apart. In clinical work, we need to consider many things and do more gene testing to know if a child has WITKOS.

Future research should focus on understanding SIN3 A better. We need to know how it works in the nervous system and how it interacts with other genes. Also, we should develop better treatments for WITKOS, like targeted drugs or gene therapy. At the same time, we need to improve how we monitor and manage WITKOS patients and make doctors and people know more about this disease to help patients live better.

In conclusion, This case is the first report of WITKOS caused by a large - fragment deletion in infancy, which expands the understanding of the genetic mechanism and early manifestations of the disease. It fills the gap in the onset of infancy, suggests the characteristics of early symptoms, helps in earlier identification and intervention, and changes the disease process. Meanwhile, it enriches the gene - phenotype association study, facilitates the precise location of the pathogenic region of the SIN3 A gene, and provides a theoretical basis for treatment. Our research indicates that for this particular case, clinical observation and genetic testing were crucial for diagnosis. Through these means, the patient's condition was identified, and subsequent rehabilitation training was implemented. The patient seemingly benefited from these interventions, with potential improvements in prognosis and quality of life observed. Nevertheless, it is essential to recognize that these conclusions are drawn from a single case and cannot be generalized. Future research with larger sample sizes is needed to validate these initial findings.

## Data Availability

Not applicable.

## Abbreviations

WITKOS: Witteveen - Kolk Syndrome
SIN3 A: Switch - insensitive 3 transcription regulator family member A
ID: Intellectual disability
MRI: Magnetic Resonance Imaging
NICU: Neonatal Intensive Care Unit
SD: Standard deviation
PGD: Preimplantation Genetic Diagnosis
DCD: Developmental Coordination Disorder
ASD: Autism Spectrum Disorder
HDAC: Histone deacetylase
HID: Histone interaction domain
CMA: Chromosome Microarray Analysis
qPCR: Quantitative Polymerase Chain Reaction
PGD: Pre - implantation Genetic Diagnosis
CNV: Copy Number Variation
UCSC: University of California, Santa Cruz
hg19: Human Genome Build 19
PCR: Polymerase Chain Reaction
NR: Not Reported
G2P2: Gravida 2 Para 2
ABO: A blood group system
